# John Fletcher

**Published:** 1836-07

**Authors:** 


### OBITUARY.

### JOHN FLETCHER, M.D.

For the chief part of the following brief sketch we are indebted to a notice published in *The Edinburgh Evening Courant* of May 26th. We have added a few particulars from our own personal knowledge and recollections of Dr. Fletcher. We have reason to believe that the original notice in the Edinburgh paper is from the pen of Dr. Lewins, of Leith.

Dr. Fletcher was the eldest son of the late Mr. Thomas Fletcher, a respectable merchant in London. It was the intention of his father to bring him up to his own profession; and Dr. Fletcher, after having enjoyed the benefit of a liberal classical education, was actually placed in the counting-house for some time. But to a mind like his a mercantile life was intolerable, and no prospect of ultimate advantage was considered by him sufficient to induce him to forego the gratification which he promised himself in the cultivation of science and literature. To these he by degrees entirely devoted himself, and at an early period gave abundant promise that, in due time, he would gain for himself a name and a fame amongst the learned of the age.

Attracted by the superior advantages which the medical school of Edinburgh presented, he came to Edinburgh in the autumn of 1813, and commenced the study of medicine; having previously attended, though irregularly, the lectures of the late Mr. Abernethy and Sir Charles Bell in London.

In 1816, Dr. Fletcher obtained the degree of doctor of medicine, after writing and publicly defending an inaugural dissertation, "De Rei Medicæ Vicissitudinibus," which, from its intrinsic excellence, but especially from the uncommon purity of its Latinity, attracted the notice, and we believe obtained the approbation, of the late distinguished Dr. Gregory, then professor of the practice of medicine in the university of Edinburgh.

Dr. Fletcher intended to settle in London, but an event occurred soon after he finished his medical studies, which not only frustrated his intentions in regard to his proposed place of residence, but entirely altered the whole plan of his future life. This event, to which it is unnecessary here more particularly to advert, deprived him of all his patrimony, and rendered it necessary that he should call his talents into operation to provide for his immediate wants; wants which, although nurtured in affluence, he could, to his credit, make few, when prudence or necessity required such a sacrifice.

The system of teaching medicine, and the mode of granting medical degrees, at the period to which we allude, was in many respects faulty and imperfect. The practice of conducting all the examinations in the Latin language made it necessary for the candidates to employ a class of men known by the name of *grinders*, who frequently did little else for their pupils than enable them to answer questions by rote in bad Latin. Dr. Fletcher's knowledge of this discreditable practice induced him to return to Edinburgh, with the view of establishing a system of tuition akin to that which is practised at Oxford in the way of private tutorship; a mode of life more congenial to his literary habits than the drudgery of general medical practice.

As soon as it was known that he had arrived in Edinburgh with the view just mentioned, the most respectable medical students flocked to him for instruction, and Dr. Fletcher was thus at once enabled to render his superior medical and classical attainments available, and for all immediate purposes to supply his loss of fortune.

The labours which devolved upon him soon became very considerable. His pupils were usually divided into classes of four, and each class had an hour's examination. His occupation began at eight o'clock in the morning, and sometimes lasted twelve hours, particularly at the close of the winter session, when the pupils were preparing for examination. To those enabled by their previous education fully to profit by his instruction, his critical commentaries on Celsus were invaluable. Few, we think, can turn to the fourth book without remembering the acuteness and judgment by which his remarks were characterised, and by which his pupils were led to a full appreciation of one of the most elegant and judicious of the ancient writers on physic and surgery. The margin of our own copy is full of notes that recall to us hours of profitable and delightful study, under his most able direction. His care was not confined to imbuing the student with a love of the classical beauties of Celsus, or to supplying him with an abundant and expressive vocabulary of Latin conversation, although in this he had perhaps no equal: his pupils felt that, under his tuition, all their previous knowledge became arranged and available. They learnt both the degree of their acquirements and the extent of their deficiencies. He required exact thoughts in exact words; and, whilst the taste was improved, the memory and understanding were vigorously exercised. We speak of him as we knew him sixteen years ago; and those who remember him about the same period will smile when they recall the magisterial authority enjoyed by so young a teacher as he then was over pupils of various ages, and even over some who were his seniors. He was systematic in every thing. His time was accurately divided, and he never wasted any portion of it. It was a maxim with him that whatever was said, written, or done, might be said, written, or done, in a certain assigned space, and that all waste of space and time was an abandonment of power that any one might exercise. His own powers were certainly tasked to the utmost, and, even at the time of which we are speaking, his health had begun to be impaired, although he endeavoured, by riding on horseback daily, to counteract the fatal tendency of too great daily exertion. In fact, when it is recollected that to each of his pupils the hour passed with Dr. Fletcher was, of all hours of the day, that of the greatest mental activity, it may be supposed that the tutor's mind was far too much exerted.

In society, Dr. Fletcher was cheerful, and even animated; he was fond of music, possessed considerable knowledge of the fine arts, and varied literary attainment. The Epistles of Rubus, (Rubi Epistolæ Edinburgenses,) published by him when a student, sufficiently attest his vivacity and wit, as well as his unlimited command of Latin and Greek. Their composition would have done honour to a university in which classical acquirement was held in greater esteem; and, with the exception of some occasional satire, the remarks they contain on the life and manners of the students, and the merits of the professors, will render them ever valuable to every lover of Edinburgh.

In the year 1822, he embodied several of his critical observations on the colloquial employment of the Latin language, in a very useful little book entitled Horæ Subsecivæ. This publication, and the Epistles of Rubus, are, we believe, now somewhat scarce; and we cannot but recommend those who desire to see something more correct in daily use than the ordinary language of prescription, to place both these little works on the shelves of their library.

Dr. Fletcher joined the Argyle-square Medical School in 1828-29, as lecturer on physiology, and latterly also lectured there on medical jurisprudence. He taught both of these branches of medical science in a manner which has seldom been equalled, never surpassed in Britain.

The rapid extension of his fame in the medical and scientific world affords unquestionable evidence of his superior attainments, whilst the steady increase of the number of his pupils proved how highly his talents as a public teacher were appreciated and valued.

In the beginning of the present year, at the urgent solicitation of several individuals who knew the extent of his attainments, he announced his intention of delivering a course of popular lectures on physiology, which he did to a numerous and intelligent audience, amongst whom were some of the most talented members of the Scottish bar and of the English church, and many other gentlemen distinguished for their intellectual endowments. The variety and extent of interesting information which Dr. Fletcher communicated,—the vast store of scientific knowledge which he brought to bear on the subject,—and the beautiful preparations and diagrams (all the work of his own hands, and which would have done credit to a first-rate artist,) by which he illustrated his subject, delighted and astonished his audience. Little, alas! did they think, whilst listening to his graphic description of the wondrous structure of organized bodies, and his luminous but delicate exposition of the functions of their various complicated organs, so illustrative, as he justly expressed it, of the wisdom and goodness of God, that his sun was to set so suddenly, whilst it was yet day, and before he had finished the work so energetically and auspiciously begun.

Dr. Fletcher, whose health for some time previously had been in a delicate state, found it necessary to confine himself to the house on the 3d of May; but so insidiously did the disease which was destined in a few days to number him with the dead make its attack, and continue its fatal progress, that no alarm had been excited in his own mind, or in that of his affectionate wife, until Dr. John A. Robertson, having occasion to call on business, discovered the actual and alarming condition of his valued friend.

Dr. Fletcher was afterwards seen by other highly talented members of the medical profession, who most anxiously and perseveringly rendered all the assistance which their art was capable of affording, but in vain. He expired early on the morning of the 10th of May, in the 44th year of his age, after a week's confinement to the house, and scarcely an entire day to his bed.

The immediate cause of Dr. Fletcher's death was an inflammatory affection of the lungs. Subsequent investigation, however, discovered that the condition of these important organs was such as to preclude the probability, if not possibility, of long life; but it is too true that Dr. Fletcher's intense and unremitting application to study was the means of shortening his valuable life.

Dr. Fletcher was the author of several works of considerable talent, but we shall here only advert to that on Physiology; and on it alone his claim to professional distinction may be safely founded. Of it only two parts are published, —the first on Organism, and the second on Life as manifested in Irritation. The third part, on Life as manifested in Sensation and Thought, has yet to appear. Although the manuscript of that part is perhaps not exactly in the state in which the lamented author would have sent it to the press, yet it is fortunately sufficiently perfect for publication, and will appear in due time.

Of the great merits of Dr. F.'s principal work, the Rudiments of Physiology, we shall speak at length in our next Number.

We cannot omit here to advert to Dr. Fletcher's published introductory discourse to his popular lectures on Physiology, which were cut short by his untimely death; a production of great talent, and strikingly characteristic of an original and independent mind. This lecture was printed at the special request of several gentlemen, well qualified to judge, who heard it delivered, and who were of opinion that its publication "in such a form as to render it easily accessible to all classes of the community would greatly subserve the cause of popular enlightenment."

It were an easy and a grateful duty to expatiate on Dr. Fletcher's private worth, on the refinement of his mind, the extent and versatility of his talents and acquirements, his high sense of honorable conduct, the value of his friendship, and the exemplary manner in which he discharged his duties in private life; but, as it is only consistent in this place to delineate his public character, we conclude this imperfect biographical sketch by the expression of our unaffected sorrow on having to record the unexpected death of a physician whose learning and whose scientific acquirements reflected honour upon medicine;—to whose instructions, too, (the writer of this notice would gratefully add,) we consider ourselves indebted for very important assistance in studies strictly professional, as well as in others from which has proceeded the happiness of many leisure hours. The reflection that Dr. Fletcher's useful life was shortened by his intellectual labours takes nothing from the deep regret we cannot but feel that his valuable exertions were not permitted to be prolonged.

